# Ethyl 1-benzyl-3-(4-fluoro­phen­yl)-1*H*-pyrazole-5-carboxyl­ate

**DOI:** 10.1107/S1600536811002340

**Published:** 2011-01-29

**Authors:** Zhe Han, Hong-Liang Zheng, Xue-Lei Tian

**Affiliations:** a Key Laboratory for Liquid-Solid Structural Evolution and Processing of Materials (Ministry of Education), Shandong University, Jinan 250061, People’s Republic of China

## Abstract

In the title compound, C_19_H_17_FN_2_O_2_, the pyrazole ring makes dihedral angles of 4.57 (16) and 81.19 (18)° with the fluoro­phenyl and benzene rings, respectively.

## Related literature

For the applications of nitro­gen-containing heterocyclic compounds in agrochemical and pharmaceutical fields, see: Ge *et al.* (2009*a* ▶,*b* ▶). For related structures, see: Ge *et al.* (2007*a* ▶,*b* ▶). 
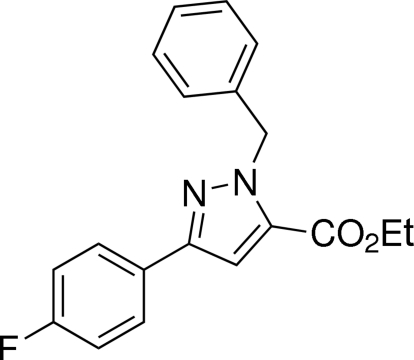


## Experimental

### 

#### Crystal data

C_19_H_17_FN_2_O_2_
*M* *_r_* = 324.35
Triclinic, 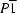

*a* = 8.119 (8) Å
*b* = 10.173 (6) Å
*c* = 10.814 (6) Å
α = 108.672 (15)°
β = 102.567 (16)°
γ = 91.460 (16)°
*V* = 821.5 (10) Å^3^
*Z* = 2
Mo *K*α radiation
μ = 0.09 mm^−1^
*T* = 298 K
0.21 × 0.16 × 0.12 mm

#### Data collection

Bruker SMART CD area-detector diffractometer
Absorption correction: multi-scan (*SADABS*; Bruker, 1997 ▶) *T*_min_ = 0.981, *T*_max_ = 0.989
4034 measured reflections
2844 independent reflections
2014 reflections with *I* > 2σ(*I*)
*R*_int_ = 0.030

#### Refinement

*R*[*F* ^2^ > 2σ(*F* ^2^)] = 0.084
*wR*(*F* ^2^) = 0.245
*S* = 1.07
2844 reflections
217 parameters
H-atom parameters constrained
Δρ_max_ = 0.51 e Å^−3^
Δρ_min_ = −0.30 e Å^−3^

### 

Data collection: *SMART* (Bruker, 1997 ▶); cell refinement: *SAINT* (Bruker, 1997 ▶); data reduction: *SAINT*; program(s) used to solve structure: *SHELXS97* (Sheldrick, 2008 ▶); program(s) used to refine structure: *SHELXL97* (Sheldrick, 2008 ▶); molecular graphics: *SHELXTL* (Sheldrick, 2008 ▶); software used to prepare material for publication: *SHELXTL*.

## Supplementary Material

Crystal structure: contains datablocks I, global. DOI: 10.1107/S1600536811002340/jh2256sup1.cif

Structure factors: contains datablocks I. DOI: 10.1107/S1600536811002340/jh2256Isup2.hkl

Additional supplementary materials:  crystallographic information; 3D view; checkCIF report

## Figures and Tables

**Fig. 1.**
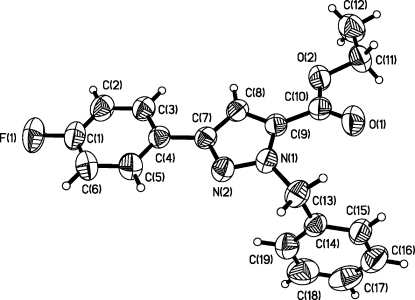
The molecular structure of (I), showing displacement ellipsoids drawn at the 50% probability level.

## supplementary crystallographic information

### Comment

Synthesis of nitrogen-containing heterocyclic compounds has been a subject of great interest due to the wide application in agrochemical and pharmaceutical fields (Ge *et al.*; 2007*a*, 2007*b*, 2009*a*, 2009*b*). Some pyrazole derivatives which belong to this category have been of interest for their biological activities. We report here the crystal structure of the title compound, (I) (Fig. 1)

### Experimental

A mixture of ethyl 3-(4-fluorophenyl)-1*H*-pyrazole-5-carboxylate (0.02 mol), benzyl chloride (0.0024 mol) and potassium carbonate (0.02 mol) in acetonitrile (100 ml) was heated to reflux for 10 h. The solvent was removed under reduced pressure and an product was isolated by column chromatography on silica gel (yield 76%). Crystals of (I) suitable for X-ray diffraction were obtained by slow cooling of the refluxed solution of the product in ethyl acetate at room temperature for 2 d.

### Refinement

All H atoms were placed in geometrically calculated positions and refined using a riding model with C—H = 0.97 Å (for CH_2_ groups) and 0.96 Å (for CH_3_ groups), their isotropic displacement parameters were set to 1.2 times (1.5 times for CH_3_ groups) the equivalent displacement parameter of their parent atoms.

### Crystal data

**Table:**

C_19_H_17_FN_2_O_2_	*Z* = 2
*M**_r_* = 324.35	*F*(000) = 340
Triclinic, *P*1	*D*_x_ = 1.311 Mg m^−^^3^
Hall symbol: -P 1	Mo *K*α radiation, λ = 0.71073 Å
*a* = 8.119 (8) Å	Cell parameters from 1645 reflections
*b* = 10.173 (6) Å	θ = 2.4–27.3°
*c* = 10.814 (6) Å	µ = 0.09 mm^−^^1^
α = 108.672 (15)°	*T* = 298 K
β = 102.567 (16)°	Block, white
γ = 91.460 (16)°	0.21 × 0.16 × 0.12 mm
*V* = 821.5 (10) Å^3^	

### Data collection

**Table:**

Bruker SMART CD area-detector diffractometer	2844 independent reflections
Radiation source: fine-focus sealed tube	2014 reflections with *I* > 2σ(*I*)
graphite	*R*_int_ = 0.030
phi and ω scans	θ_max_ = 25.0°, θ_min_ = 2.1°
Absorption correction: multi-scan (*SADABS*; Bruker, 1997)	*h* = −9→6
*T*_min_ = 0.981, *T*_max_ = 0.989	*k* = −12→11
4034 measured reflections	*l* = −12→12

### Refinement

**Table:**

Refinement on *F*^2^	Primary atom site location: structure-invariant direct methods
Least-squares matrix: full	Secondary atom site location: difference Fourier map
*R*[*F*^2^ > 2σ(*F*^2^)] = 0.084	Hydrogen site location: inferred from neighbouring sites
*wR*(*F*^2^) = 0.245	H-atom parameters constrained
*S* = 1.07	*w* = 1/[σ^2^(*F*_o_^2^) + (0.165*P*)^2^] where *P* = (*F*_o_^2^ + 2*F*_c_^2^)/3
2844 reflections	(Δ/σ)_max_ < 0.001
217 parameters	Δρ_max_ = 0.51 e Å^−^^3^
0 restraints	Δρ_min_ = −0.30 e Å^−^^3^

### Special details

**Table:**

Geometry. All e.s.d.'s (except the e.s.d. in the dihedral angle between two l.s. planes) are estimated using the full covariance matrix. The cell e.s.d.'s are taken into account individually in the estimation of e.s.d.'s in distances, angles and torsion angles; correlations between e.s.d.'s in cell parameters are only used when they are defined by crystal symmetry. An approximate (isotropic) treatment of cell e.s.d.'s is used for estimating e.s.d.'s involving l.s. planes.
Refinement. Refinement of *F*^2^ against ALL reflections. The weighted *R*-factor *wR* and goodness of fit *S* are based on *F*^2^, conventional *R*-factors *R* are based on *F*, with *F* set to zero for negative *F*^2^. The threshold expression of *F*^2^ > σ(*F*^2^) is used only for calculating *R*-factors(gt) *etc*. and is not relevant to the choice of reflections for refinement. *R*-factors based on *F*^2^ are statistically about twice as large as those based on *F*, and *R*- factors based on ALL data will be even larger.

### Fractional atomic coordinates and isotropic or equivalent isotropic displacement parameters (Å^2^)

**Table:**

	*x*	*y*	*z*	*U*_iso_*/*U*_eq_	
F1	0.0446 (3)	0.7940 (3)	1.0242 (2)	0.0941 (8)	
O1	0.3457 (3)	0.4182 (3)	0.1271 (2)	0.0856 (8)	
O2	0.3605 (2)	0.2792 (2)	0.2513 (2)	0.0633 (6)	
N1	0.2763 (3)	0.6363 (2)	0.3527 (2)	0.0524 (6)	
N2	0.2347 (3)	0.7094 (3)	0.4667 (2)	0.0544 (6)	
C1	0.0891 (4)	0.7506 (4)	0.9042 (3)	0.0659 (9)	
C2	0.1320 (4)	0.6187 (4)	0.8574 (3)	0.0681 (9)	
H2	0.1317	0.5585	0.9063	0.082*	
C3	0.1762 (4)	0.5763 (3)	0.7355 (3)	0.0604 (8)	
H3	0.2066	0.4865	0.7023	0.072*	
C4	0.1760 (3)	0.6651 (3)	0.6618 (3)	0.0486 (7)	
C5	0.1315 (4)	0.7985 (3)	0.7127 (3)	0.0620 (8)	
H5	0.1308	0.8594	0.6642	0.074*	
C6	0.0878 (4)	0.8422 (4)	0.8360 (3)	0.0694 (9)	
H6	0.0584	0.9321	0.8710	0.083*	
C7	0.2232 (3)	0.6190 (3)	0.5320 (3)	0.0476 (7)	
C8	0.2607 (3)	0.4883 (3)	0.4600 (3)	0.0507 (7)	
H8	0.2623	0.4084	0.4842	0.061*	
C9	0.2947 (3)	0.5017 (3)	0.3453 (3)	0.0496 (7)	
C10	0.3367 (3)	0.3994 (3)	0.2303 (3)	0.0560 (8)	
C11	0.3982 (4)	0.1676 (3)	0.1414 (3)	0.0707 (9)	
H11A	0.5020	0.1943	0.1210	0.085*	
H11B	0.3066	0.1471	0.0614	0.085*	
C12	0.4175 (5)	0.0438 (4)	0.1851 (4)	0.0898 (12)	
H12A	0.5120	0.0638	0.2615	0.135*	
H12B	0.4370	−0.0338	0.1131	0.135*	
H12C	0.3160	0.0211	0.2091	0.135*	
C13	0.3159 (3)	0.7111 (3)	0.2662 (3)	0.0595 (8)	
H13A	0.2573	0.7947	0.2804	0.071*	
H13B	0.2741	0.6528	0.1730	0.071*	
C14	0.5029 (3)	0.7517 (3)	0.2919 (3)	0.0532 (7)	
C15	0.5833 (4)	0.7185 (3)	0.1874 (3)	0.0629 (8)	
H15	0.5221	0.6684	0.1006	0.075*	
C16	0.7528 (5)	0.7588 (4)	0.2109 (5)	0.0802 (11)	
H16	0.8057	0.7374	0.1397	0.096*	
C17	0.8439 (4)	0.8301 (4)	0.3378 (5)	0.0899 (13)	
H17	0.9591	0.8567	0.3533	0.108*	
C18	0.7659 (5)	0.8628 (4)	0.4434 (5)	0.0916 (12)	
H18	0.8287	0.9107	0.5302	0.110*	
C19	0.5959 (4)	0.8248 (4)	0.4209 (3)	0.0698 (9)	
H19	0.5431	0.8480	0.4921	0.084*	

### Atomic displacement parameters (Å^2^)

**Table:**

	*U*^11^	*U*^22^	*U*^33^	*U*^12^	*U*^13^	*U*^23^
F1	0.0974 (14)	0.131 (2)	0.0598 (13)	0.0193 (13)	0.0397 (11)	0.0254 (13)
O1	0.126 (2)	0.0790 (17)	0.0587 (15)	0.0083 (14)	0.0397 (14)	0.0212 (13)
O2	0.0740 (13)	0.0592 (13)	0.0566 (13)	0.0104 (10)	0.0235 (10)	0.0135 (11)
N1	0.0546 (12)	0.0581 (15)	0.0503 (14)	0.0089 (10)	0.0189 (10)	0.0215 (12)
N2	0.0537 (12)	0.0584 (15)	0.0572 (15)	0.0104 (10)	0.0201 (11)	0.0225 (13)
C1	0.0529 (16)	0.092 (2)	0.0498 (18)	0.0047 (15)	0.0175 (13)	0.0157 (18)
C2	0.0730 (19)	0.081 (2)	0.055 (2)	0.0028 (16)	0.0184 (16)	0.0284 (18)
C3	0.0653 (17)	0.0633 (19)	0.0573 (18)	0.0065 (14)	0.0194 (14)	0.0233 (16)
C4	0.0415 (13)	0.0544 (16)	0.0481 (16)	0.0016 (11)	0.0106 (11)	0.0149 (14)
C5	0.0641 (17)	0.066 (2)	0.060 (2)	0.0139 (14)	0.0200 (14)	0.0230 (17)
C6	0.0698 (18)	0.074 (2)	0.064 (2)	0.0205 (16)	0.0261 (16)	0.0144 (19)
C7	0.0412 (12)	0.0525 (16)	0.0491 (16)	0.0030 (11)	0.0098 (11)	0.0178 (14)
C8	0.0479 (13)	0.0551 (18)	0.0507 (17)	0.0010 (11)	0.0126 (12)	0.0195 (14)
C9	0.0434 (13)	0.0542 (17)	0.0481 (16)	0.0010 (11)	0.0101 (11)	0.0137 (13)
C10	0.0517 (15)	0.0607 (19)	0.0514 (18)	−0.0028 (12)	0.0121 (13)	0.0137 (15)
C11	0.083 (2)	0.066 (2)	0.058 (2)	0.0123 (16)	0.0230 (16)	0.0080 (18)
C12	0.117 (3)	0.065 (2)	0.087 (3)	0.018 (2)	0.033 (2)	0.019 (2)
C13	0.0616 (16)	0.070 (2)	0.0578 (18)	0.0117 (14)	0.0170 (14)	0.0345 (16)
C14	0.0622 (15)	0.0529 (17)	0.0557 (18)	0.0109 (12)	0.0219 (13)	0.0278 (15)
C15	0.0761 (19)	0.0630 (19)	0.061 (2)	0.0124 (14)	0.0303 (16)	0.0270 (16)
C16	0.080 (2)	0.081 (2)	0.103 (3)	0.0177 (19)	0.048 (2)	0.045 (2)
C17	0.060 (2)	0.086 (3)	0.139 (4)	0.0027 (18)	0.028 (2)	0.057 (3)
C18	0.081 (2)	0.088 (3)	0.095 (3)	−0.0153 (19)	0.005 (2)	0.029 (2)
C19	0.078 (2)	0.073 (2)	0.056 (2)	−0.0005 (16)	0.0187 (16)	0.0186 (17)

### Geometric parameters (Å, °)

**Table:**

F1—C1	1.362 (4)	C9—C10	1.456 (4)
O1—C10	1.208 (4)	C11—C12	1.480 (5)
O2—C10	1.325 (4)	C11—H11A	0.9700
O2—C11	1.451 (4)	C11—H11B	0.9700
N1—N2	1.340 (3)	C12—H12A	0.9600
N1—C9	1.360 (4)	C12—H12B	0.9600
N1—C13	1.460 (3)	C12—H12C	0.9600
N2—C7	1.339 (3)	C13—C14	1.504 (4)
C1—C2	1.358 (5)	C13—H13A	0.9700
C1—C6	1.360 (5)	C13—H13B	0.9700
C2—C3	1.379 (4)	C14—C15	1.380 (4)
C2—H2	0.9300	C14—C19	1.385 (4)
C3—C4	1.382 (4)	C15—C16	1.371 (5)
C3—H3	0.9300	C15—H15	0.9300
C4—C5	1.382 (4)	C16—C17	1.360 (6)
C4—C7	1.470 (4)	C16—H16	0.9300
C5—C6	1.392 (4)	C17—C18	1.378 (6)
C5—H5	0.9300	C17—H17	0.9300
C6—H6	0.9300	C18—C19	1.372 (5)
C7—C8	1.388 (4)	C18—H18	0.9300
C8—C9	1.374 (4)	C19—H19	0.9300
C8—H8	0.9300		
			
C10—O2—C11	116.1 (3)	O2—C11—H11A	110.3
N2—N1—C9	111.5 (2)	C12—C11—H11A	110.3
N2—N1—C13	118.3 (2)	O2—C11—H11B	110.3
C9—N1—C13	129.6 (2)	C12—C11—H11B	110.3
C7—N2—N1	105.6 (2)	H11A—C11—H11B	108.5
C2—C1—C6	122.8 (3)	C11—C12—H12A	109.5
C2—C1—F1	119.1 (3)	C11—C12—H12B	109.5
C6—C1—F1	118.1 (3)	H12A—C12—H12B	109.5
C1—C2—C3	118.4 (3)	C11—C12—H12C	109.5
C1—C2—H2	120.8	H12A—C12—H12C	109.5
C3—C2—H2	120.8	H12B—C12—H12C	109.5
C2—C3—C4	121.1 (3)	N1—C13—C14	112.9 (2)
C2—C3—H3	119.5	N1—C13—H13A	109.0
C4—C3—H3	119.5	C14—C13—H13A	109.0
C5—C4—C3	118.9 (3)	N1—C13—H13B	109.0
C5—C4—C7	120.4 (3)	C14—C13—H13B	109.0
C3—C4—C7	120.7 (3)	H13A—C13—H13B	107.8
C4—C5—C6	120.3 (3)	C15—C14—C19	119.2 (3)
C4—C5—H5	119.8	C15—C14—C13	120.6 (3)
C6—C5—H5	119.8	C19—C14—C13	120.1 (3)
C1—C6—C5	118.5 (3)	C16—C15—C14	120.3 (3)
C1—C6—H6	120.7	C16—C15—H15	119.8
C5—C6—H6	120.7	C14—C15—H15	119.8
N2—C7—C8	110.7 (2)	C17—C16—C15	120.3 (3)
N2—C7—C4	119.9 (2)	C17—C16—H16	119.9
C8—C7—C4	129.3 (3)	C15—C16—H16	119.9
C9—C8—C7	105.6 (3)	C16—C17—C18	120.1 (3)
C9—C8—H8	127.2	C16—C17—H17	120.0
C7—C8—H8	127.2	C18—C17—H17	120.0
N1—C9—C8	106.6 (2)	C19—C18—C17	120.2 (4)
N1—C9—C10	122.5 (3)	C19—C18—H18	119.9
C8—C9—C10	130.9 (3)	C17—C18—H18	119.9
O1—C10—O2	123.3 (3)	C18—C19—C14	119.9 (3)
O1—C10—C9	125.5 (3)	C18—C19—H19	120.1
O2—C10—C9	111.1 (3)	C14—C19—H19	120.1
O2—C11—C12	107.1 (3)		
			
C9—N1—N2—C7	−1.2 (3)	C13—N1—C9—C10	−9.9 (4)
C13—N1—N2—C7	−173.5 (2)	C7—C8—C9—N1	−0.2 (3)
C6—C1—C2—C3	0.0 (5)	C7—C8—C9—C10	−177.9 (3)
F1—C1—C2—C3	179.8 (3)	C11—O2—C10—O1	−0.3 (4)
C1—C2—C3—C4	−0.4 (5)	C11—O2—C10—C9	178.2 (2)
C2—C3—C4—C5	0.4 (4)	N1—C9—C10—O1	−6.4 (4)
C2—C3—C4—C7	180.0 (3)	C8—C9—C10—O1	171.0 (3)
C3—C4—C5—C6	0.0 (4)	N1—C9—C10—O2	175.1 (2)
C7—C4—C5—C6	−179.6 (2)	C8—C9—C10—O2	−7.4 (4)
C2—C1—C6—C5	0.4 (5)	C10—O2—C11—C12	−179.2 (3)
F1—C1—C6—C5	−179.4 (3)	N2—N1—C13—C14	94.7 (3)
C4—C5—C6—C1	−0.4 (5)	C9—N1—C13—C14	−76.1 (4)
N1—N2—C7—C8	1.0 (3)	N1—C13—C14—C15	128.1 (3)
N1—N2—C7—C4	−178.9 (2)	N1—C13—C14—C19	−52.8 (4)
C5—C4—C7—N2	4.6 (4)	C19—C14—C15—C16	−0.8 (4)
C3—C4—C7—N2	−175.0 (2)	C13—C14—C15—C16	178.3 (3)
C5—C4—C7—C8	−175.3 (3)	C14—C15—C16—C17	1.1 (5)
C3—C4—C7—C8	5.0 (4)	C15—C16—C17—C18	−0.4 (6)
N2—C7—C8—C9	−0.5 (3)	C16—C17—C18—C19	−0.6 (6)
C4—C7—C8—C9	179.4 (2)	C17—C18—C19—C14	0.9 (6)
N2—N1—C9—C8	0.8 (3)	C15—C14—C19—C18	−0.2 (5)
C13—N1—C9—C8	172.1 (3)	C13—C14—C19—C18	−179.3 (3)
N2—N1—C9—C10	178.8 (2)		
